# Isothermal Nucleic Acid Amplification Technologies for the Detection of Equine Viral Pathogens

**DOI:** 10.3390/ani11072150

**Published:** 2021-07-20

**Authors:** Alexandra Knox, Travis Beddoe

**Affiliations:** Department of Animal, Plant and Soil Sciences and Centre for AgriBioscience La Trobe University, Bundoora, VIC 2082, Australia; a.knox@latrobe.edu.au

**Keywords:** equine, viruses, loop-mediated isothermal amplification, insulated isothermal polymerase chain reaction, field-deployable, point-of-care testing

## Abstract

**Simple Summary:**

Equine viral diseases remain a prominent concern for human and equine health globally. Many of these viruses are of primary biosecurity concern to countries that import equines where these viruses are not present. In addition, several equine viruses are zoonotic, which can have a significant impact on human health. Current diagnostic techniques are both time consuming and laboratory-based. The ability to accurately detect diseases will lead to better management, treatment strategies, and health outcomes. This review outlines the current modern isothermal techniques for diagnostics, such as loop-mediated isothermal amplification and insulated isothermal polymerase chain reaction, and their application as point-of-care diagnostics for the equine industry.

**Abstract:**

The global equine industry provides significant economic contributions worldwide, producing approximately USD $300 billion annually. However, with the continuous national and international movement and importation of horses, there is an ongoing threat of a viral outbreak causing large epidemics and subsequent significant economic losses. Additionally, horses serve as a host for several zoonotic diseases that could cause significant human health problems. The ability to rapidly diagnose equine viral diseases early could lead to better management, treatment, and biosecurity strategies. Current serological and molecular methods cannot be field-deployable and are not suitable for resource-poor laboratories due to the requirement of expensive equipment and trained personnel. Recently, isothermal nucleic acid amplification technologies, such as loop-mediated isothermal amplification (LAMP) and insulated isothermal polymerase chain reaction (iiPCR), have been developed to be utilized in-field, and provide rapid results within an hour. We will review current isothermal diagnostic techniques available to diagnose equine viruses of biosecurity and zoonotic concern and provide insight into their potential for in-field deployment.

## 1. Introduction

Since their domestication, equines have been a pivotal part of history and continue to provide fundamental economic value worldwide [1,2,3]. With an estimated global population of over 59 million domesticated horses [4], the global equine industry is valued at approximately USD $300 billion annually [2,5]. The industry comprises of two main categories: primary equine activities and secondary equine activities. Primary activities are defined as sectors directly involved with equines, such as horse trainers, coaches, breeders, professional competitors and jockeys, and clubs and associations. In contrast, the secondary sector is for services that are indirectly involved with equines, or provide external services for equine owners, such as equine health professionals, and support industries including transport and sale of horses [6]. These sectors provide essential services for countries worldwide, significantly contributing to strong economic growth, particularly in developing communities [3,7].

In addition to the economic contributes, the global equine industry has an estimated 1.6 million full-time employees. In particular, the racing industry is the major contributor with significant levels of employment, from trainers and jockeys to breeders [8]. With over 160,000 races held worldwide annually [9], the economic substance of this industry is apparent. Additionally, the racing industry provides longstanding culture and traditions throughout the world. For example, the Melbourne Cup, held in Australia, is the most renowned handicap Thoroughbred equine racing event of the year [10]. Over 22 countries participate and import their Thoroughbreds to Australia for the racing seasons, reaching a yearly global audience of over 700 million [11].

While the equine industry is extremely important economically and socially, as either organized equine sport or companion animals, there is a range of zoonotic and non-zoonotic viral infections that are harmful to both equine and human health [12,13,14]. For example, Australia experienced an outbreak of equine influenza in 2007, affecting roughly 69,000 horses and resulting in a significant economic loss estimated at a current AUD $571 million, with eradication alone costing an inflated $370 million [15]. Fortunately, Australia was able to eradicate this virus; however, further worldwide viral outbreaks continuously loom over the fate of the industry [14]. With continuous global movement, importation and subsequent housing of large equine populations increasing worldwide, it is essential to increase biosecurity measures and diagnostics against viral diseases to avoid rapid transmission and spread [16].

Moreover, many of these diseases do not have effective treatment options; thus, there is an increased demand to control and eradicate diseases through improved biosecurity protocols [12,17,18]. The ability to accurately diagnose diseases early could lead to better management and treatment strategies [16]. Diagnostic methods have been developed over previous decades due to advances in biochemistry, molecular biology, and immunology research [19] and continue to improve presently. These advancements, such as and point-of-care (POC) diagnostics, are increasingly utilized and sought after for routine diagnosis for equine viral infections [16]. While many molecular tests, such as polymerase chain reaction (PCR), have been developed to detect equine viral infections, they are not field-deployable, thus are unable to support rapid decision-making for disease control and treatment [20,21,22]. To overcome these drawbacks current research has moved toward isothermal nucleic amplification techniques, such as loop-mediated isothermal amplification (LAMP) [23] and insulated isothermal polymerase chain reaction (iiPCR) [24]. Both these methods utilize an enzymatic reaction to amplify nucleic acid, at a constant temperature [23,24]. LAMP and iiPCR have been previously demonstrated to be field deployable POC diagnostic techniques, achieving results in less than an hour. These powerful tools have been extensively researched for equine medicine, and continue to pave the way for newer, more accessible diagnostic methods [23,24,25,26,27,28,29]. Here we review the field-deployable technology, LAMP and iiPCR, and their application to diagnose equine viral infections.

## 2. Equine Viral Diseases of Biosecurity Concern

Despite strict global import and biosecurity policies, infectious disease outbreaks continue to occur globally, particularly with equine viruses [13,30]. These outbreaks have detrimental effects on the equine’s health and welfare and inhibit their regular activity, subsequently harming the industry’s economy in the associated geographical regions [15,17,20]. The World Organisation of Animal Health (OIE) releases a yearly report stating the diseases of concern for terrestrial animals, which includes equine viral pathogens [31,32]. This section outlines each of these viral diseases.

### 2.1. African Horse Sickness

African horse sickness (AHS) is a non-contagious arthropod-borne virus widely distributed across sub-Saharan Africa [33]. There are four forms of the disease: subclinical, subacute or cardiac, acute respiratory, and mixed. Mortality rates vary with disease severity, with the mixed and acute respiratory forms having the highest mortality rates at 70–80% and 95%, respectively [34]. As AHS is transmitted to a susceptible host via a mosquito vector, mainly *Culicoides* species, the virus can quickly spread before containment [35,36]. Moreover, recent studies have warned that the distribution of AHS is expanding from endemic areas to regions with suitable climatic environments that are home to other mosquito species which share ancestry with *Culicoides* species [35,36,37]. In fact, four horses in Thailand during March 2020 tested positive for AHS after succumbing to infection just 12–24 h after initially displaying symptoms, making quick diagnosis paramount [38]. Furthermore, the government had to quickly implement control measures and utilize live attenuated vaccines [39]. Despite the availability and the continuous development of AHS vaccines [40], many countries including Australia, still do not have approval for implementation to these options [41], leaving them vulnerable to a potential outbreak without a means to control the disease [36].

### 2.2. Equine Encephalomyelitis (Western)

While western equine encephalitis (WEE) persistence has been declining considerably since the mid-20th century [42,43,44,45], this arbovirus remains on the OIE list of notifiable diseases [31,32]. The choice to continually survey for this virus is attributed to the potential for further significant and detrimental outbreaks [42]. The virus circulates in an enzootic cycle between mosquitoes, specifically *Culex* species, and passerine birds. However, infection of humans and equines can occur in the event of a spillover during peak vector activity periods [42,46,47,48]. Cases have declined since the 1940s and 1950s, which saw peak cases in humans and equines in America’s western region [42]. Clinical signs in horses start with biphasic fever, followed by a range of neurological and behavioural symptoms, including anorexia, ataxia, aggression, somnolence, aimless wandering, general depression, and animals eventually succumb to the disease [49,50,51]. In humans, WEE infections can result in neurological sequelae post-infection which places a severe strain on health care system. Treatment costs for human infection varies between $21,000 and $3 million per case [42,52]. There is no specific antiviral treatment for both humans and equines, with supportive care the only available option [48,51,53]. The recommended diagnostic techniques for WEE include virus isolation and reverse-transcription PCR (RT-PCR) [31]; however, development is in progress for a nucleic acid sequence-based amplification (NASBA) assay that could provide a more rapid means of detection and be used for field samples [54]. However, this assay is yet to be validated.

### 2.3. Equine Infectious Anaemia

Equine infectious anaemia (EIA) is a non-contagious disease of equids; however, equines and ponies are more susceptible to severe clinical infection of this virus. This globally prevalent disease causes all infected equids to become life-long carriers [55,56,57,58]. Transmission occurs through blood-feeding vectors, specifically horseflies and deerflies, blood-contaminated fomites, and in utero via transplacental transmission [55,59,60]. Clinical signs vary depending on the strain virulence and susceptibility of the equid host. Majority of cases occur in three phases; the acute or initial phase, followed by a chronic phase, and finally the inapparent, or long-term asymptomatic phase [55,57,61]. Clinical symptoms typically appear within seven to thirty days post-infection, with fever, depression, and possible thrombocytopenia; however, signs may be mild and can be overlooked, resulting in misdiagnosis or underreporting [55,57,62]. Equines experience reoccurring episodes of fever, increased heart and respiratory rates, anaemia, muscle weakness, and loss of condition for around one year following initial infection [55]. Equines will then become chronic life-long carriers with no apparent symptoms [57]. EIA has caused severe outbreaks throughout Europe and has re-emerged in countries after multiple years of disease absence [63]. Diagnostics are exclusively performed by serological techniques, including enzyme-linked immunosorbent assay (ELISA) [59,61,64]; however, only the agar gel immunodiffusion (AGID) assay remains OIE approved [31,59]. Despite this recommendation, the AGID assay can require a secondary test for validation [31] and is not appropriate for equines in the acute phase of infection as viral load is too low [65].

### 2.4. Equine Influenza

Equine influenza (EI) is reported as the most important globally distributed respiratory disease in equines [66]. The fatality rates are contributed to the secondary bacterial infection; however, the prognosis typically relies on the individual immune status [67]. EI is highly contagious and has multiple transmission pathways, including contaminated fomites. Furthermore, there is no specific treatment, and despite an available vaccine, significant outbreaks continue to occur [66]. As previously stated, Australia experienced an EI outbreak in 2007 that lasted for five months affecting roughly 69,000 horses [15,68]. The magnitude of the outbreak affected 9,600 properties, including companion equine households, business incomes, and horse associations [69]. The strict biosecurity measures were implemented and remain ongoing; however, a recent survey of 1,224 horse owners directly involved in the 2007 outbreak reported that 32% of participants were not in favor of continuous biosecurity measures. More concerningly, approximately 30% of participants had low biosecurity compliance, stating they implemented biosecurity procedures “not often” or “never” [30]. This complacency, or lack of understanding, further enhances the risk of outbreaks throughout the equine industry [70]. More recently, the United States has had waves of annual epidemics in 2015, 2016, and 2017, affecting 23, 16, and 22 states, respectively [66].

Additionally, in 2018, Chile experienced a re-emergence of the H3N8 EI strain, which had not previously been detected since 2012. Further genetic testing confirmed that this virus had high homology with other viruses that had been in circulation in Europe and Asia [71]. It is apparent that EI is continuously present almost globally, and outbreaks will continue to fluctuate without adequate means of rapid diagnostics to quickly and efficiently intervene [66,70,72].

### 2.5. Equine Viral Arteritis

Equine viral arteritis (EVA) significantly impacts the breeding sector of the equine industry, as the disease affects both the respiratory and reproductive status of the animal. EVA incidences have been increasing over the past 20 years [73]. While the majority of cases are subclinical, serious long-term effects cause significant production losses [74]. The disease is rarely fatal in healthy horses; however, 50%–60% of infected pregnant mares can experience abortion [75,76]. In addition, stallions can be long-term carriers while remaining asymptomatic [74]. Like many other equine viral diseases, there is no affective treatment, restricting countries to rely on biosecurity measures [77]. EVA can be spread through venereal mechanisms; leaving breeding programs at a high transmission risk, which is a prominent industry in many countries. Long-term carrier stallions may have to undergo castration to prohibit accidental transmission to mares, and removing them from breeding programs [77,78]. The disease has already caused a reduction in commercial value of horses, with higher costs for breeding and commercialization of semen and embryos [77]. This was particularly evident in 2007 when France experienced an outbreak of EVA due to the distribution of infected semen, causing the disease to spread into 17 premises. It was suspected that horizontal transmission occurred via farm employees. This outbreak was deemed the most significant of its kind, with considerable economic disruptions [79]. While the use and advocation of vaccine programs can help alleviate the burden, persistently importing infected equines remains highly problematic for vulnerable countries [73].

### 2.6. Equine Rhinopneumonitis (Caused by EHV-1)

Equine rhinopneumonitis caused by equine herpesvirus type 1 (EHV-1) is globally distributed, particularly in regions with a significant equine presence [80,81,82]. Additionally, equine rhinopneumonitis can be caused by equine herpesvirus type 4 (EHV-4), furthering exacerbating the prevalence of this disease [80,83,84]. Despite the availability of both live and inactivated vaccines for EHV-1, the persistence of this virus remains [85,86]. While transmission is predominantly via the respiratory route, contact or ingestion of contaminated fomites or contact through foetuses or placenta of an infected mare is possible [81,82,85,87]. Due to the inapparent respiratory clinical signs, it is often misdiagnosed as other viral or bacterial diseases, leaving equine populations susceptible to the introduction of the virus [82,88].

Additionally, younger equines appear to be highly susceptible to infection, with 80%–90% of animals less than two years old carrying this respiratory disease [89]. While non-steroidal anti-inflammatory drugs (NSAIDs) may assist in elevating symptoms, there is no specific cure for disease elimination [85,89]. Despite the sporadic recovery from infection, horses can often develop a secondary infection that can be fatal [90]. Despite the development of a PCR diagnostic assay, virus isolation is still required for comparative analysis to other diseases, making accurate disease identification laborious [31,84].

## 3. Zoonotic Equine Viral Diseases of Concern

### 3.1. Hendra Virus

Hendra virus (HeV) is a well-documented zoonotic equine virus that has been a prominent concern for the equine industry [91]. While this emerging, highly transmissible virus is exclusively isolated to Australia, it has caused several outbreaks and is predominantly fatal. The primary vector of HeV is fruit bats; although the exact mechanism of transmission to equines is not fully understood. However, it is thought that equines potentially consume contaminated fruit bat droppings via their feed. Transmission among equines and subsequently to humans is through either direct (via secretions) or indirect (via fomites) routes [92]. The disease presents as influenza-like symptoms with rapid deterioration [93]. There is no specific treatment or cure for HeV, and progression can lead to septic pneumonia, and more recently found, encephalitis [91,94]. In 2008, five equines and two human infection cases occurred in Queensland, Australia. As a result, many veterinary clinics had to close due to the ramifications of acquiring the virus [95]. Despite the currently available vaccine targeting equine HeV, there is no vaccine available for humans, leaving all equine industry personnel vulnerable to infection. The suggested prevention for human infections to avoid infected horses and maintaining personal hygiene [91,93,96]. With the limited feasibility to this approach it is reasonable to expect another HeV outbreak. An outbreak of HeV would infer severe economic losses from the cancellation of events and prohibition of animal movement [91,96].

### 3.2. Japanese Encephalitis

Typically known as a significant human neurological disease, the Japanese encephalitis virus (JeV) also infects equines with three clinical syndromes: transient, lethargic, and hyperexcitable type [97,98]. Horses infected with either transient or lethargic type typically recover within a week; however, death is common with the hyperexcitable type [99]. In addition to encephalitis, clinical signs in equines can also include a fluctuating fever, decreased appetite, jaundice and haemorrhaging in the mucous membranes, staggering, and sweating [97]. While this disease is not globally distributed, many populated countries in Asia encounter a combined 70,000 human cases per year with 10,000 of these being fatal [100]. Limited barriers separating endemic and JeV-free countries, coupled with the ease of mosquito vector transmission and limited availability of vaccines in non-endemic countries, make the risk of outbreaks significantly high [101]. Additionally, accurate detection of viral prevalence is problematic due to a short duration of viraemia and asymptomatic infections [100].

### 3.3. Ross River Virus

Ross River virus (RRV) is the most widespread and significant arbovirus in Australia and neighboring islands, such as Fiji and the Cook Islands, frequently causing large epidemics in humans and equines [102]. There has been an increase in incidences of infection across Australia due to recent flooding and climatic changes optimal to harbor the mosquito vector [103,104,105]. RRV can infect equines and humans through mosquito bites and causes various symptoms ranging from distal limb oedema and arthritis to neurological diseases [106,107,108]. Additionally, infected equines reluctantly move during infection due to debilitating joint pain, causing a significant reduction in production and performance [108]. The prescribed treatment for equines includes NSAIDs therapy considering there is no available vaccine [109]. The majority of infection reports state that recovery on average takes two to five days; however, recently prolonged recoveries of up to five months to a year have been noted. Not only is RRV a significant concern for human and equine health, the equine industry could also infer potential economic losses in the millions, attributed to restrictions on movement and trade, loss of performance in infected equines, and wastage [107]. Australia’s favorable environmental and ecological conditions have facilitated an endemic state that encounters reoccurring outbreaks; with a likelihood of climatic change enhancing the global occurrence of optimal conditions for the spread of disease. Subsequently, increased outbreaks would cause the implementation of strict biosecurity measures to ensure both human and animal welfare; resulting in restrictions on animal movement, production, and quarantining in turn causing undoubtable economic losses [106].

### 3.4. West Nile Virus

West Nile virus (WNV) is closely related to JeV; however, seldom causes encephalitis in humans and equines [110,111]. In fact, most infected humans will be asymptomatic, with only around 20% of cases resulting in influenza-like symptoms [112,113]; nevertheless, viral infections can still be fatal [114]. For equines, clinical signs can include neurological disease, such as encephalitis and ataxia, as along with the loss of appetite, depression, and, infrequently, fever [115,116,117]. While there is a vaccine available for equines [118,119], the risk for expansive transmission and cross-species spread is foreseeable, due to the broad range of hosts, such as reptiles, mammals, birds, and ticks [120]. The main transmission mechanism is via carrier mosquitoes after biting an infected host, namely the Corvidae family of birds [112]. A mosquito then can infect several animals and bird species, including equines and humans, which are incidental hosts [120]. Jointly with the ease of transmissibility, WNV has a wide geographical distribution throughout Africa, Europe, West Asia, Australia, and North America, giving a high probability of global spread [110,121]. Considering there is no specific WNV treatment, supportive care is recommended until the infection subdues, typically spontaneously [111]. Currently, detection relies on nested and real-time reverse transcription PCR (real-time RT-PCR) [31,122,123]. However serological diagnosis, such as seroconversion, is more reliable, as current molecular tools are unable to provide accurate diagnostics due to their sensitivities and the low viremia associated with WNV infections [31]. While control programs are dependent on surveillance, particularly of deceased crows [120,124] and vaccine for equines [118,119], this does not entirely protect humans [120]. Ultimately, the concurrent broad host range and vast geographical distribution of WNV has the potential for a global outbreak with significant impact [121,125].

## 4. Current Diagnostic Techniques for Equine Viral Diseases

Diagnostics in the equine industry are vital to restrict the spread of infectious diseases [19], particularly with frequent and high equine movement [16]. Due to this substantial amount of transport nationally and internationally, the OIE has provided a list of 117 diseases of concern for terrestrial animals; six of these include equine viruses (Table 1) [32]. Additionally, OIE has produced a reference guide for terrestrial animal diagnostics to promote the use of “gold-standard” testing worldwide [31]. Table 1 presents the current gold standard diagnostic techniques for each of the named equine viruses and zoonotic equine viruses of concern.

### 4.1. Serological Diagnostics

Serological assays are used for an array of diagnostics in equine medicine, including viral diseases. These assays detect antibodies of a specific infection from the serum, providing an indirect mean of diagnosis [127]. Serological analysis is commonly utilized in equine medicine for diagnostics, due to the attractive advantages they provide [16,31,127]. The use of serological assays allows for detection of samples with low quantity of antigens, visualization with the naked eye, and can also be used on a wide range of pathogens [16,127]. However, drawbacks of these assays must be considered. Firstly, many of these assays have a high probability of either false-positive or false-negative results [127]. Secondly, serological assays often are required be coupled with a secondary detection method for an official confirmation. Additionally, as with other types of diagnostics, the assays often require specialized equipment, and are time consuming and labor intensive, either due to the assay procedure or subsequently from a secondary diagnostic test for confirmation [16,128,129]. However, despite these drawbacks these assay techniques remain a well-established technique in veterinary medicine [127]. One common diagnostic technique is ELISA, an assay that detects specific immune responses with the use of antibodies, antigens, and enzymes [16]. ELISA is considered a convenient, safe, and reproducible diagnostic technique, with several different variations, such as dot-ELISA and falcon assay screening test-ELISA (FAST-ELISA). These developments have allowed for a quicker assay that is cost-efficient with results that can be easily interpretative [129]. ELISA has been proven as a reliable diagnostic tool for equine influenza, and its use is advocated by OIE [31,127]. Despite serological assays being well-established in veterinary diagnostics, many of these assays are being replaced with newer molecular technologies [16].

### 4.2. Molecular Diagnostics

Molecular diagnostics have been continuously evolving, providing more sensitive detection of nucleic acid [130]. As a result, these tools have been increasingly favored and utilized in equine medicine for clinical diagnostics. PCR has been the most advocated molecular tool, with the greatest success [16]. PCR tests can detect various organisms, including slow-growing or challenging to cultivate organisms, overcoming limitations in previously used diagnostic tools [131]. Furthermore, PCR provides other advantages such as rapid time to gain results, the sensitivity to detect smaller quantities of microorganisms and is not reliant on the host’s immune response [54,132,133].

Nevertheless, this method comes with drawbacks, including reaction inhibition from substances within samples, such as urea, varying techniques and protocols, frequency of false-negatives and false-positives, a high risk of contamination, and the requirement of expensive equipment and experienced personnel [54,134,135]. Additionally, as PCR is based on nucleic acid amplification, the results can only confirm the presence or absence of pathogenic DNA in the sample [136,137]. Yet, PCR is still considered a powerful tool that is utilized consistently in equine medicine [138]. Advancements in PCR-based technologies have been developed throughout recent years and have expanded diagnostic capabilities for detecting clinical infections, particularly for viruses [139].

Recently, PCR has been developed for real-time evaluation of results by utilizing intercalating dyes or target-specific probes [140], minimising handling of PCR products throughout the procedure, therefore reducing the risk of contamination [141]. In addition, many PCR assays have been described to utilize real-time PCR coupled with reverse-transcription, termed real-time RT-PCR [138]. Thus, this technique is now quickly replacing diagnostics that were previously [31,142,143] performed by conventional PCR.

Molecular diagnostics are a promising tool for detection of viral infections, but they can be misinterpreted by inexperienced personnel and are not applicable for in-field use or in poorly resourced laboratories, limiting their global disease surveillance application [138].

## 5. Isothermal Techniques

Isothermal techniques are driven by enzymatic reactions to amplify nucleic acid at a single temperature, thus allowing POC or field-deployable testing [144,145]. Additionally, some of these techniques do not require samples to be purified, allowing for direct use of living cells from field obtainable samples [145]. This advantage has influenced diagnostic technique development to further exploit isothermal conditions over conventional methods such as PCR, which requires various temperature cycles to complete amplification. Multiple isothermal technologies are currently available, with unique features and template types (Table 2) [146].

**Figure 1:**
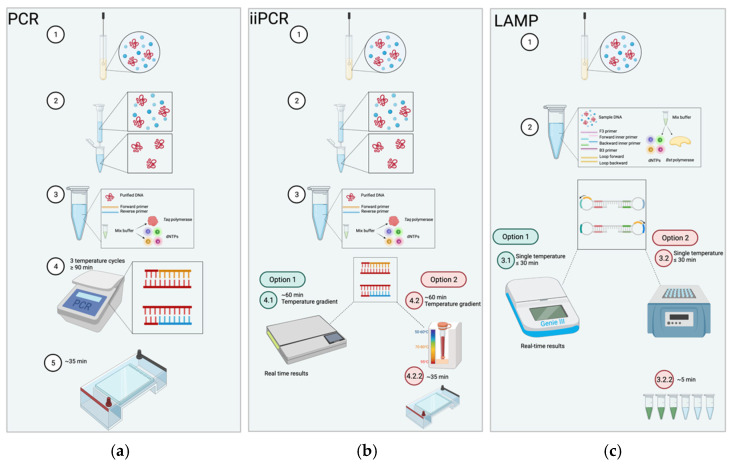
Comparison of polymerase chain reaction (PCR) insulated isothermal polymerase chain reaction (iiPCR) and loop-mediates isothermal amplification (LAMP) procedures. (**a**) PCR procedure is as follows; 1. sample is collected; 2. sample is purified; 3. contents for PCR are mixed including the purified sample, forward and reverse primers, and master mix buffer which includes *Taq* polymerase and dNTPs; 4. the reaction is ran on a thermocycler for ≥90 min cycling through three temperatures for the denaturation, annealing and extension stages; 5. PCR products are subjected to agarose gel electrophoresis for approximately 35 min at 100 amps to visualize results. (**b**) iiPCR follows a similar starting procedure to PCR where, 1. samples are collected, and 2. purified, 3. contents are mixed as such for PCR. However, reaction is conduced within capillary tubes with a copper ring at the base and lid, where the mixture is heated underneath to create a temperature gradient through convection; reactions last for around 1 h. This can be achieved through two options: 4.1. an automated portable machine, POCKIT™ (GeneReach USA, Lexington, MA, USA) where results are displayed in real time; alternatively, 4.2. an insulated box that requires the products to undergo (4.2.2) agarose gel electrophoresis for approximately 35 min at 100 amps to visualize results. (**c**) The LAMP procedure is as follows, 1. samples are collected and 2. mixed with 4–6 primers (F3, B3, forward inner primer and backward inner primer, and optional loop primers). LAMP can tolerate impurities in samples and therefore do not required to be purified. 3. The mixture is heated at a single temp temperature for typically ≤30 min. This can also be achieved by two options: 3.1. an automated machine, Genie III™, (OptiGene Horsham, Eng, UK), where results are displayed in real time; alternatively, 3.2. a heat source, such as a water bath, where products are visualized through (3.2.2) fluorescence for approximately 5 min to observe a color change. Created with BioRender.com.

Despite promising and extensive research into isothermal techniques for pathogenic detection, equine diagnostic technology for viruses has been limited to two isothermal technologies, LAMP and iiPCR (Figure 1). This is probably due to several different companies producing commercial reagents for both LAMP and iiPCR assays. This has allowed researchers to develop assays for the detection of different viruses. However, the benefits of lower costs, low energy requirement, method simplicity, and ease of field deployment of isothermal technologies justify further research for diagnostics for equine medicine [23,24,149,155,156].

## 6. Application of LAMP for Equine Viral Diseases

### 6.1. Principles of LAMP

LAMP was designed to overcome associated drawbacks of traditional serological and molecular diagnostics. Unlike other assays, LAMP does not require expensive equipment, trained personnel, laborious methods making it easily deployed in resource-poor settings [23]. This technique is relevant for various applications, such as rapid, sensitive, and specific diagnostics, genetic, and POC testing [20,21]. In addition, the DNA template does not need to be denatured, which is a requirement of conventional PCR [156], and results can be visualized with the naked eye. This reduces the number of required steps and subsequent downstream processing time and the possibility of cross-contamination, an issue common to other diagnostic techniques [157,158]. LAMP utilizes four to six primers that recognize six to eight distinct regions of a target sequence, enhancing the rapidity of the assay which is performed at a constant temperature (Figure 1) [23]. This application has proven to be a reliable diagnostic technique for a diverse range of pathogens, including equine infectious diseases [21,25,26,159,160].

### 6.2. Application of LAMP for Equine Viral Diseases

Due to the wide success of the currently available LAMP assays, there is continuous development of this technology for various applications. One such technique is incorporating a reverse-transcription to detect RNA viruses, coined RT-LAMP [27,149,161,162]. In addition to conventional LAMP, this approach has been utilized for numerous equine viral disease (Table 3).

Nemoto et al. [164] developed a LAMP assay to detect both equine herpesvirus type 1 (EHV-1) and 4 (EHV-4), as well as differentiating between the wild-type EHV-1 (ΔgE) strain, which is the non-neuropathogenic strain [169]. This assay detected glycoprotein C (gC) in both viruses for diagnostic purposes and EHV-1 glycoprotein E (gE) for distinction from the wild-type strain, which has a deletion at the gE gene. This assay reported similar sensitivity compared to PCR, but at a lower cost, and a time to positive between 30 min to 1 h when ran at a constant temperature of 60–65 °C. The results were visualized by gel electrophoresis and by eye through observation of a color change. The detection limit for EHV-1 and EHV-4 showed high sensitivity at 1 and 0.1 plaque-forming unit (pfu) per reaction, respectively, with no cross-reaction towards other viral and bacterial equine diseases. Therefore, this LAMP assay has the potential to replace current PCR diagnostic assays to accurately determine equine herpesvirus [164].

RT-LAMP was developed to detect RNA viruses, which performs synthesis of DNA for detection concurrently with amplification [149]. This technique was also adopted by Nemoto and colleagues to develop two novel assays detecting equine influenza strains H3N8 [166] and H7N7 [167]. Both assays were designed to target the HA gene of influenza from nasal swab samples acquired in the field from horses presenting with a fever (≥38 °C). The assays were specific to differentiate the separate strains. The assay was 3 to 10 times more sensitive than the commercial serological ELISA test (Espline Influenza A&B-N ELISA test (Fujirebio, Japan)).

Additionally, H3N8 RT-LAMP assay was ten times more sensitive than the previously developed RT-PCR test, while the H7N7 RT-LAMP compared the same as the RT-PCR. The H3N8 assay detected 35 additional positive samples that were not positively identified by both the RT-PCR and the Espline Influenza A&B-N test. The detection limit for H3N8 and H7N7 during RT-LAMP was 10^−5^ and 10^−4^ copies per reaction, respectively, achieved a positive threshold in roughly 60 min. The results of these assays were visually determined by turbidity, allowing for identification without specialized equipment. This approach for detection shows the simplicity that LAMP assays offer and the ability for in-field diagnostics and large-scale surveillance. The authors recommend combining these RT-LAMP assays into a panel diagnostic test to differentiate between the two strains [166,167].

Furthermore, Fowler et al. [163] utilized RT-LAMP to develop rapid detection of African horse sickness (AHS), resulting in a similar sensitivity to a previously developed AHS real-time RT-PCR. By targeting the structurally conserved VP7 gene that forms the outer capsid, the assay could detect the viral DNA within 30 min. The results were visualized by DNA intercalating dyes, contained in the reaction master mix (ISO-001, OptiGene Ltd., Horsham, UK). Despite the ease of visualization, the paper suggested adapting the assay to use real-time fluorescence for ease of application in-field, adopted from previous experiments [170,171]. To convert the assay to an in-field diagnostic technique, it was recommended to use lyophilized reagents and eliminate the RNA extraction procedure by implementing an automated extraction procedure, as utilized by Waters et al. [170] and Howson et al. [171].

In 2018, Han et al. [165] presented a preliminary study of a RT-LAMP assay for equine infectious anemia. This study employed detected the gag non-structural protein (gag nsP) of the virus, using a recombinant plasmid, pMD-19T-gag, rather than field or clinical samples. While this assay has a longer reaction time of two hours to detection 100 copies/µL, it provides a starting point for further development. As this assay only included four primers, it is possible to decrease the assay time through the use of loop primers. Promisingly, the RT-LAMP assay did not detect other pathogens, showing high specificity, which can be further validated through the testing of clinical samples. Furthermore, results were visualized through a color change, allowing for the possibility of conversion to a field deployable diagnostic technique.

Wheeler et al. [159] developed a panel of RT-LAMP assays for the detection of St. Louis encephalitis virus (SLEV) and western equine encephalitis (WEEV), which additionally incorporated a previously developed assay for WNV [149]. While a multiplex reverse transcription-quantitative polymerase chain reaction (RT-qPCR) has been developed for these viruses [172], it is not field-deployable [167]. The developed RT-LAMP targeted the non-structural protein 4 (nsP4) gene for WEEV, and the 3′ untranslated region (3′-UTR) for SEEV, and had a detection limit the same as the previously developed WNV RT-LAMP at 0.1 pfu per reaction [149,159]. Despite having a sensitivity marginally less than the previously developed RT-qPCR assay, both the SLEV and WEEV assays were performed in less than 30 min [159], and WNV RT-LAMP in under 17 min [149], supremely faster than the RT-qPCR assay. As this panel assay was performed on mosquitos in the field, it can be deployed as a large-scale surveillance program and as a rapid diagnostic technique [149,159].

Additionally, Foord et al. [26] developed a LAMP assay that was able to detect the conserved P-gene of Hendra virus before clinical signs appeared. This assay also compared utilizing a lateral flow device (LFD) to agarose gel electrophoresis for visual detection. While the LFD was not as sensitive in comparison to the gel, it was able to show results in five minutes, providing further confirmation of LAMP’s field deployable abilities. Furthermore, the LAMP assay was able to detect additional positive results that was previously deemed “indeterminate” using a TaqMan assay. The authors suggest the simple procedure allow for LAMP to be employed in resource-poor environments. In addition, the capability of detecting positive cases prior to the onset of symptoms is ideal for critical situations, such as a Hendra virus outbreak, that require immediate results.

These developed assays show that both LAMP and RT-LAMP can be performed in-field as POC diagnostic technique. However, while various LAMP assays have been developed to detect viruses of concern to equines, both with high sensitivity and specificity, excluding the WNV RT-LAMP assay [149,159], none of these assays has been commercialized. The reasoning for this lack of commercially available assays remains unclear.

## 7. Application of iiPCR for Equine Viral Diseases

### 7.1. Principles of iiPCR

iiPCR is a recently developed assay involving an isothermal convective device [24]. The technique amplifies nucleic acids like PCR; however, it replaces the use of an expensive thermocycler with a simpler, portable, insulated device that consists of a copper ring attached to polycarbonate capillary tubes (R-tube™) underneath (Figure 1). The thermal convective device allows for reagents to proceed through gradient temperatures within the single tube [173], thus performing the required denaturation, annealing, and extension steps in a portable manner. Additionally, the insulation protects the assay from environmental influence, permitting its use in the field [24]. iiPCR has been analyzed as more sensitive than RT-PCR, achieving results within 1 h [155] through simple and cost-effective procedures [29,173,174].

### 7.2. Applications of iiPCR for Equine Viral Diseases

The iiPCR technique has been implemented for several equine viral diseases (Table 4). As seen in RT-LAMP, reverse-transcription has been integrated with iiPCR (iiRT-PCR) to detect RNA viruses through the generation of amplified cDNA [155].

Advancements of this technique have resulted in developing a portable machine that allows for automatic detection, termed POCKIT™ Nucleic Acid Analyzer by GeneReach USA (GeneReach USA, Lexington, MA, USA). This lightweight machine detects amplicons using hydrolysis technology recognizing fluorescent signals [173]. Carossino et al. [155] utilized this technology to develop a iiRT-PCR assay to detect EVA. This assay reported to have significant accuracy with a detection limit of 10 copies per reaction in one hour. Furthermore, compared to a previously developed RT-qPCR diagnostic test for EVA [178], the iiRT-PCR assay was ten-fold more sensitive. Therefore, this iiRT-PCR further exhibited the potential of future assays to be exploited in field for POC diagnostics.

Additionally, the assay did not encounter inhibition when using tissue samples that had been previously observed with the developed RT-qPCR assay [155]. The robustness of iiPCR and iiRT-PCR assays are advantageous as promising alternatives for diagnostic and control implementation [155,175,176]. However, despite numerous successful assays developed for equine infectious diseases, commercially available kits using the POCKIT™ Nucleic Acid Analyzer (GeneReach USA, Lexington, MA, USA) have been restricted to the aquaculture industry. Thus, for further traction of this technique and technology, commercialization should be made applicable to the equine industry.

## 8. Future Applications of LAMP and iiPCR for Equine Viral Diseases

Field deployable and POC assays for disease detection are becoming increasingly sought after [179,180,181], particularly for livestock and large animals to avoid transport-related stress and cost [182,183,184]. These portable diagnostic techniques allow for sampling and testing to take place pen-side or in the field without a laboratory [183], subsequently eliminating the transportation process and providing results in real-time for immediate treatment and control of infectious diseases [185,186]. Both LAMP and iiPCR are ideal for field-deployable diagnostics owing to their robustness, cost-efficiency, accessibility, and portable instruments, and are advantageous over conventional PCR assays (Table 5) [181,187,188].

Both LAMP and iiPCR have portable machines that are lightweight (roughly 5 kg), robust, and only require an AC voltage or car battery to operate [189]. In addition, these machines are paving the way for newer diagnostic techniques, providing countless opportunities for alternative diagnostic technologies [181,188].

It should be noted that the feasibility of these assays is still dependent on sampling techniques and preparations [189]. Comparability, LAMP can tolerate sample impurities and inhibitory substances as it has been developed to eliminate nucleic acid extraction steps altogether [190,191], whereas iiPCR still requires nucleic acid purification [189]. In addition, inhibitors of PCR, can interfere with the assay results rendering them inaccurate and often false-negative outcomes. Therefore, sampling techniques that are in-field appropriate is a rapidly expanding area of research. Research groups have developed extraction systems that could isolate total nucleic acids through column-based methods. Despite these methods being described as user-friendly [192], contamination and degradation of RNA was still an issue, attributed to extensive manual handling throughout sample processing [189]. Thus, a field-deployable fully automated extraction system was developed by GeneReach, coined the Taco™ mini extraction system [193]. This machine can handle an array of samples, including more complex tissue and swab samples, to completely extract nucleic acids from up to eight samples concurrently. This magnetic beads-based tool is relatively inexpensive, compacted, and lightweight allowing for immediate use and practical storage after use [155,177,189,193]. However, this process adds an extra 45 min of processing to the assay, and while inexpensive, additional machinery is not suited to resource-poor facilities. The more intricate nucleic acid extraction requirements of iiPCR are hindering its POC application in comparison to the practical LAMP.

## 9. Conclusions

With an estimated value of USD $300 billion annually, involving more than 59 million domestic horses and 1.6 million full-time employees, it is essential to protect the global equine industry from disease outbreaks. Despite strict worldwide biosecurity procedures, the threat of a viral outbreak, including zoonotic diseases, remains imminent. Due to the increasing amount of national and international movement and subsequent dense housing of horse populations, the spread of viral diseases could be rapid and devastating, particularly with asymptomatic carriers. Current “gold-standard” diagnostic techniques, such as serological and molecular technology, remain prominent within the industry; however, they come with several drawbacks that limit their use, particularly in resource-poor settings. Newer isothermal techniques, such as LAMP and iiPCR, allow for rapid diagnosis and offer the opportunity to be field-deployable. However, further research is required to ultimately eliminate laborious procedures, particularly in nucleic acid extraction. While LAMP has been developed to tolerate sample impurities and does not require extraction steps, iiPCR continues to rely on extra machinery to provide an automated extraction technique. Nevertheless, the use of these methodologies remains advantageous over traditional methods for POC testing, based on their rapidity, sensitivity, specificity, and inexpensiveness. Thus, there is strong reasoning to develop new diagnostics using isothermal technology as alternatives to traditional techniques for rapid disease identification and quick implementation of control measures.

## Figures and Tables

**Table 1:** OIE [32] notifiable equine viruses and zoonotic viruses of biosecurity concern with their prescribed “gold-standard” diagnostic tests for confirmation of disease [31].

Disease	Prescribed Diagnostic Test/s [31]
OIE listed notifiable equine viral diseases [32]	
African horse sickness	RT-PCR ^1^ Virus isolation
Equine encephalomyelitis (Western)	RT-PCR Virus isolation
Equine infectious anaemia	AGID ^2^
Equine influenza	ELISA ^3^ RT-PCR
Equine viral arteritis	CF ^4^ PCR VN ^5^ Virus isolation
Equine viral rhinopneumonitis (EHV-1)	PCR VN Virus isolation
Zoonotic equine viral diseases of concern	
Hendra virus	RT-PCR Virus isolation
Japanese encephalitis	RT-PCR Virus isolation
Ross River virus	RT-PCR [126] Virus isolation [126]
West Nile virus	RT-PCR

^1^ Reverse-transcription polymerase chain reaction, ^2^ agar gel immunodiffusion assay, ^3^ enzyme-linked immunosorbent assay, ^4^ complement fixation, ^5^ virus neutralization.

**Table 2:** Summary of developed isothermal techniques.

Technique	Template	Temperature ^1^	Enzyme	Reference
Helicase-dependent amplification (HDA)	DNA	65 °C	Helicase	[147]
Insulated isothermal PCR (iiPCR)	DNA	95 °C	Taq DNA polymerase	[24]
Insulated isothermal reverse-transcription PCR (iiRT-PCR)	RNA	95 °C	Taq DNA polymerase M-MLV reverse transcription	[148]
Loop-mediated isothermal amplification (LAMP)	DNA	65 °C	*Bst* DNA polymerase	[23]
Reverse transcription loop-mediated isothermal amplification (RT-LAMP)	RNA	65 °C	*Bst* DNA polymerase AMV reverse transcription	[149]
Multiple displacement amplification (MDA)	DNA	30 °C	Φ29 DNA polymerase	[150]
Nucleic acid sequence-based amplification (NASBA)	RNA	50 °C	T7 RNA polymerase RNase H AMV reverse transcription	[151]
Rolling circular amplification (RCA)	DNA	30 °C	Phi29 *Bst* DNA polymerase Vent *exo*-DNA polymerase T7 RNA polymerase	[152]
Recombinase polymerase amplification (RPA)	DNA RNA	37 °C	DNA polymerase	[153]
Strand displacement amplification (SDA)	DNA	60 °C	DNA polymerase	[154]

^1^ Average temperature used in respective assays.

**Table 3:** Current LAMP assays developed for equine viral diseases.

Disease	Type	Vector-Borne	Target Gene	Sample	Detection Limit	In-Field	Ref
African horse sickness	dsRNA	Yes—Midges, Mosquito	Vp7	Horse—Blood	n/a	Yes	[163]
Equine herpesvirus 1	dsDNA	No	Glycoprotein C	Horse—Nasal swab ^1^	1 pfu/rxn	No ^1^	[164]
			Glycoprotein E	Horse—Nasal swab ^1^	1 pfu/rxn		
Equine herpesvirus 4	dsDNA	No	Glycoprotein C	Horse—Nasal swab ^1^	1 pfu/rxn	No ^1^	[164]
Equine infectious anaemia	ssRNA	Yes—Horse and deer flies	Gag nsP	Recombinant plasmid	0.1 pfu/rxn	No	[165]
Equine influenza (H3N8)	ssRNA	No	HA	Horse—Nasal swab	10^−5^ copies/rxn	Yes	[166]
Equine influenza (H7N7)	ssRNA	No	HA	Horse—Nasal swab	10^−4^ copies/rxn	Yes	[167]
Equine coronavirus	ssRNA	No	Nucleocapsid	Horse—Nasal swab, fecal samples	10^1.8^ copies/rxn	Yes	[168]
Hendra virus	ssRNA	No	P	Horse—Nasal swab ^1^	10^−5^ copies/rxn	No ^1^	[26]
St Louis encephalitis	ssRNA	Yes—mosquito	UTR	Mosquito	<0.1 pfu/rxn	Yes	[159]
Western equine encephalitis	ssRNA	Yes—mosquito	nsP4	Mosquito	100 pfu/ml	Yes	[159]
West Nile virus	ssRNA	Yes—mosquito	E	Mosquito	0.1 pfu/ml	Yes ^2^	[149]

^1^ Experimentally infected animals, ^2^ secondary experiment.

**Table 4:** Current LAMP assays developed for equine viral diseases.

Disease	Type	Vector-Borne	Target Gene	Sample	Detection Limit	In-Field	Ref
Equine viral arteritis	ssRNA	No	ORF7	Horse—Tissue, semen	10 copies/rxn	Yes	[155]
Equine herpesvirus 3	dsDNA	No	gG	Horse—Perineal and genital swabs	6 copies/rxn	Yes	[175]
Equine herpesvirus myeloencephalopathy (EHM) caused by EHV-1	dsDNA	No	ORF3	Horse—Tissue	13 copies/rxn	Yes	[176]
Equine infectious anaemia	ssRNA	Yes—Horse and deer flies	5′ UTR Exon 1 of *tat* gene	Horse—Tissue	8 copies/rxn	Yes	[177]
Equine influenza (H3N8)	ssRNA	No	HA	Horse—Nasal swab	11 copies/rxn	Yes	[148]

**Table 5:** Comparison of conventional PCR to iiPCR and LAMP assays and procedures.

Properties	PCR	iiPCR	LAMP
Temperature	Cycles through 3 temperatures 55–95 °C	Constant temperature drives temperature gradient 15–30 °C	Constant temperature 60–65 °C
Equipment	Thermocycler	Specialized reaction tube Fluorescence-based detector	Heat source
Field-deployable	No	Yes	Yes
Reaction time	At least 90 min	≤60 min	<30 min
Sensitivity	Starts at nanograms	Starts at nanograms	Starts at femtograms
Specificity	Requires specific primer design Prone to errors	Requires specific primer design Prone to errors	Tolerates combination of primer designs
Visualization	Only through gel electrophoresis	Real-time available	Real-time available
Template prep	Requires purification	Requires purification	Tolerates impurities
Cost	$$$	$$	$
